# Chiral Plasmonic Filtering in Silver Nanowires via Point‐Defect‐Induced Mode Conversion and Interference

**DOI:** 10.1002/nap2.70324

**Published:** 2026-11-05

**Authors:** Huijie Wang, Yifan Chen, Wei Dai, Wenjun Zhang, Wenqiang Wang, Li Chen, Zhiqiang Guan, Hongxing Xu

**Affiliations:** ^1^ School of Physics and Technology Center for Nanoscience and Nanotechnology, and Key Laboratory of Artificial Micro and Nanostructures of Ministry of Education Wuhan University Wuhan China; ^2^ Institute of Physics Henan Academy of Sciences Zhengzhou China; ^3^ Institute of Light Resources and Environmental Science Henan Academy of Sciences Zhengzhou China; ^4^ Henan Academy of Sciences Zhengzhou China

**Keywords:** interference effect, mode conversion, on‐chip plasmon filtering, point defects, silver nanowires

## Abstract

Silver nanowire plasmonic waveguides offer subwavelength light confinement for integrated photonic circuits. Typically, chiral surface plasmon polariton control depends on excitation polarization. Here, we introduce a nanogroove point defect as a new regulatory dimension to realize on‐chip chiral filtering. Using finite‐difference time‐domain simulations together with scattering matrix method, we show that the nanogroove selectively couples the TM_0_ and HE_−1_ modes, and the filtering stems from constructive or destructive interference between direct transmission and mode‐converted pathways. With optimized geometry (depth: 45 nm, width: 55 nm), a chiral extinction ratio of 2.77 is achieved. Experimentally, a nanogroove fabricated via focused ion beam milling exhibits a measured chiral extinction ratio of 1.96, in good agreement with theoretical predictions. Furthermore, the scattering matrix method allows systematic investigation of the influence of defect number and spacing on the filtering performance, predicting an enhanced ratio of ∼3.7 for two optimally spaced defects. This work demonstrates point‐defect engineering as a compact, design‐ready strategy for on‐chip plasmonic devices.

## Graphical abstract

A single nanogroove milled into a silver nanowire is shown to function as an on‐chip chiral plasmonic filter. The point defect selectively converts and interferes with guided plasmonic modes, enabling handedness‐dependent transmission under ± 45° polarized excitation. This defect‐engineering approach introduces a new regulatory dimension beyond conventional polarization control for integrated nanophotonic circuits.

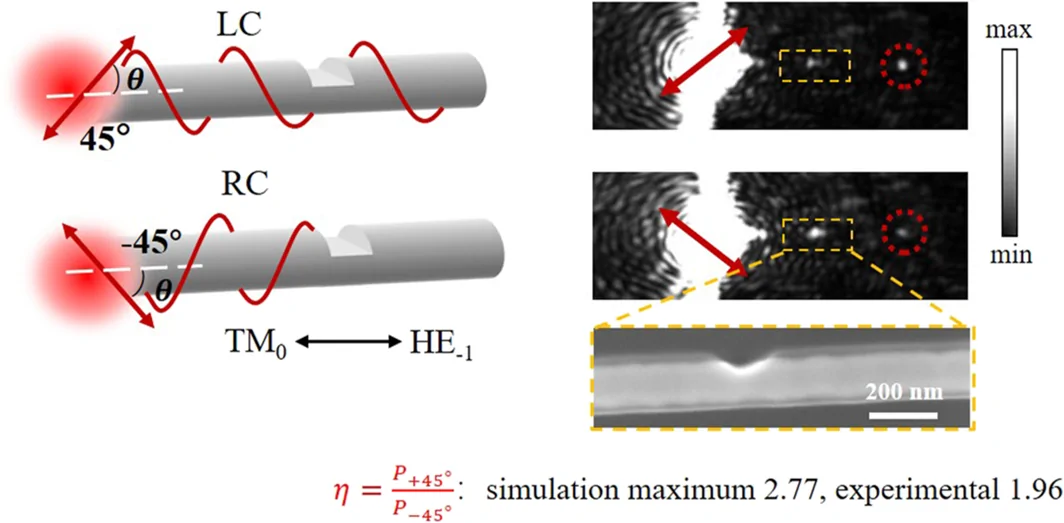

## 1 Introduction

The nanoscale guidance and manipulation of light are fundamental to advancing next‐generation optical communication and information processing technologies. Surface plasmon polaritons (SPPs), electromagnetic waves arising from the collective oscillation of free electrons at metal‐dielectric interfaces enable optical confinement and manipulation beyond the diffraction limit, offering a promising route to miniaturize optoelectronic circuits [1, 2, 3, 4, 5]. Compared to conventional silicon photonics, plasmonic elements inherently support larger bandwidths and higher operational speeds, making them attractive candidates for high‐speed on‐chip interconnects [6, 7]. Over the past decades, various plasmonic waveguide geometries have been explored, including metal films, stripes, slots, and nanowires [8, 9, 10]. Among these, silver nanowires (Ag NWs) have emerged as a particularly versatile and widely studied platform, supporting low‐loss, subwavelength wave guiding with multimodal capabilities. Significant progress has been made in both fundamental understanding and functional applications, such as optimizing propagation characteristics [11], probing spin–orbit and Raman interactions [12, 13], and modulating excitonic interconversion [14], which collectively advance the development of on‐chip photodetectors [15] and nanolasers [16, 17].

Chemically synthesized Ag NWs typically support several guided plasmonic modes with well‐defined near‐field profiles and distinct symmetries. The selective excitation and modal control of these SPPs can be achieved through several means, such as engineering the local dielectric environment [18], controlling the incident polarization [19, 20], utilizing scattering from adjacent nanoparticles [10], or exploiting resonance effects within a Fabry–Pérot cavity [21, 22]. This tunability has led to the demonstration of precise functional operations such as cascaded logic gates [23], multi‐channel routing [24, 25, 26], and high‐visibility quantum interference [27] within assembled NW networks. However, many of these approaches depend on multi‐component architectures requiring nanometric alignment [28] or complex lithographically‐defined geometries, such as channel drop filters [29], metal slot [8], MIM and tooth‐shaped waveguides [30, 31], or rely on elaborately modulated surface structures for broadband filtering [32, 33, 34, 35]. A more direct and deterministic method to locally control the near‐field distribution and modal interference within a single, continuous nanowire, which is a capability critical for precise and high‐fidelity signal manipulation in integrated plasmonic circuits, has remained a challenge.

In this work, we report a new strategy that moves beyond excitation polarization control. We introduce a single nanogroove, milled as a point defect into Ag NWs, to enable chiral selective filtering of propagating SPPs. Here “chiral” refers to the handedness of the helical near‐field distribution, rather than structural chirality of the nanowire. The underlying physics is comprehensively analyzed using a combined approach of finite‐difference time‐domain (FDTD) simulations and scattering matrix method for a two‐port system. This framework enables a detailed analysis of how the defect induces mode conversion between fundamental eigenmodes and modulates their complex amplitudes. We systematically optimize the defect position, geometry, and quantity for the chiral filtering application and explain the origin of the achievable chiral extinction ratio (up to ∼3.7). Finally, we experimentally verify the chiral filtering functionality via both far‐field scattering and fluorescence imaging on a fabricated Ag NW. Beyond conventional approaches, this nanogroove‐based scheme provides a deterministic, nanofabrication‐enabled method for plasmonic filtering. Consequently, this work opens a novel perspective for designing on‐chip integrated polarization‐sensitive devices, such as compact polarizers and beam splitters, advancing the toolkit for functional nanophotonic circuits.

## 2 Results and Discussions

### 2.1 Chiral Filtering Effect of a Single Nanogroove: FDTD Simulation and Phenomenon

To demonstrate the principle of chiral filtering via a point defect, we performed full‐wave three‐dimensional FDTD simulations. As schematically illustrated in Figure 1a, a linearly polarized Gaussian beam at a wavelength of 632.8 nm is focused onto one end of Ag NW with a diameter of 120 nm, embedded in a homogeneous dielectric medium (refractive index *n* = 1.5). Chiral SPPs arise from coherent superposition of guided modes. Specifically, this superposition involves the three fundamental guided modes supported by the NW: the axially symmetric transverse magnetic mode (TM_0_) and the degenerate first‐order hybrid electric modes (HE_1_ and HE_−1_), whose field distributions are shown in Figure 1b.

**FIGURE 1:**
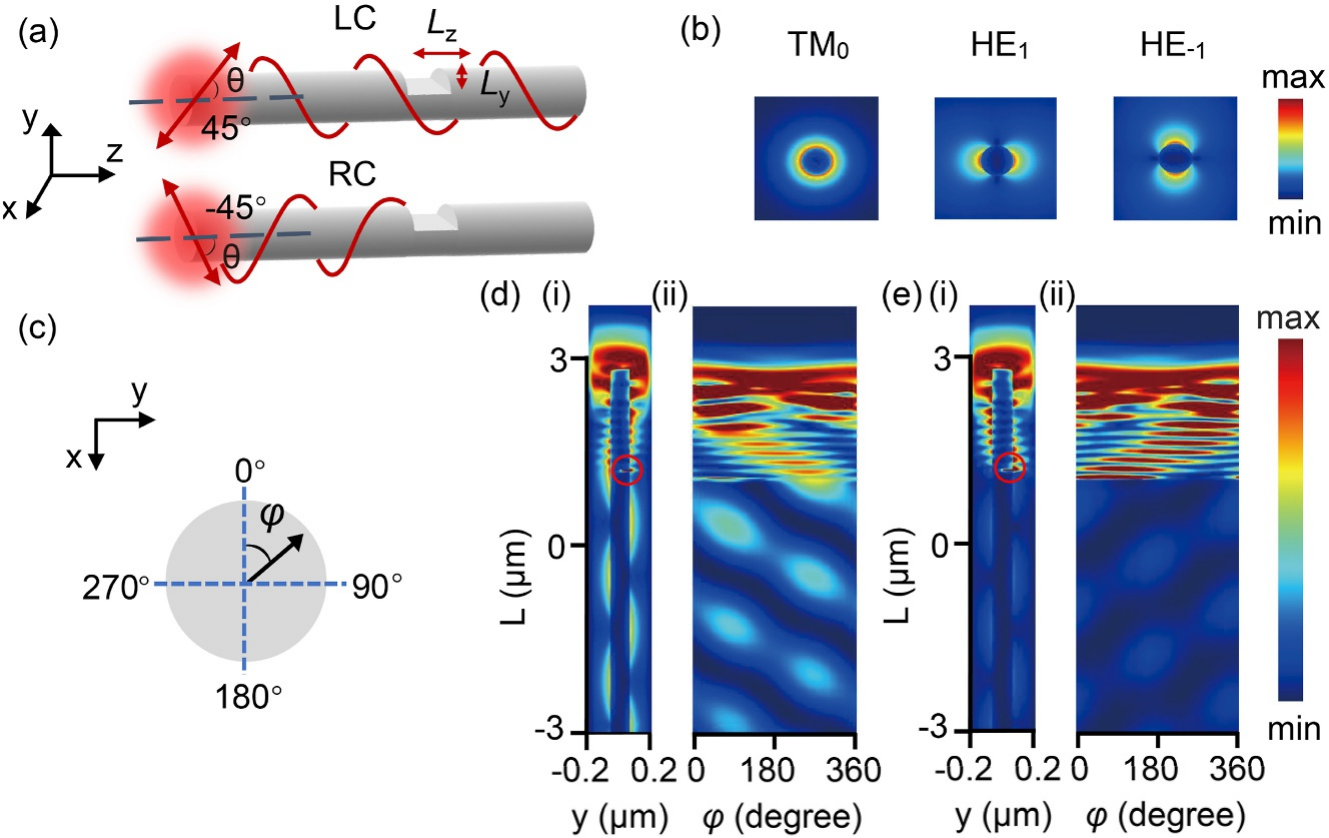
Simulated chiral filtering by a single nanogroove on a silver nanowire (Ag NW). (a) Schematic of the filtering concept. Chiral SPPs are launched by focusing ± 45° polarized light onto the NW end. A single nanogroove, positioned 1.75 μm away, selectively transmits or suppresses the SPPs based on their handedness. LC: Left‐handed chirality passes through. RC: Right‐handed chirality is blocked. The angles *θ* between the polarization direction of the incident light and the *z‐*axis are 45° and −45°, respectively. The dimensions of the nanogroove are set as $L_{y}=45\;\text{nm}$ and $L_{z}=55\;\text{nm}$. (b) Electric field distributions of the three fundamental guided modes: TM_0_, HE_1_, and HE_−1_. (c) Definition of the coordinate system and the azimuthal unfolding used for field analysis. (d, e) Cross‐sectional (left) and unfolded azimuthal (right) electric field distributions under −45° (d) and +45° (e) polarized excitation. The red circles indicate the position of the nanogroove. A clear contrast in transmission past the defect is observed.

A single rectangular nanogroove, acting as a point defect, is introduced at a distance of 1.75 μm from the excitation end. This location corresponds to a field maximum for SPPs launched by −45° polarized light. Figure 1d,e show the simulated electric field distributions in a cross‐sectional plane and their unfolded azimuthal plots for +45° and −45° excitations, respectively. The dimensions of the nanogroove are set as $L_{y}=45\;\text{nm}$ (depth along the *y‐*axis) and $L_{z}=55\;\text{nm}$ (width along the *z‐*axis). A striking contrast in transmission is observed. After passing the nanogroove, the SPPs excited with +45° polarization maintain a high intensity, whereas those excited with −45° polarization are significantly suppressed. This clear differential transmission establishes the nanogroove's functionality as a chiral plasmonic filter based on the handedness of the incoming SPPs. The handedness of the excited SPPs is further quantified by the circular polarization degree *C* (Supporting Information S1: Figure S1), which reaches approximately 0.8 for a three‐groove configuration (included here as an upper‐bound estimate of the achievable handedness; the single‐groove case exhibits a similar trend), confirming the chiral nature of the propagation.

### 2.2 Scattering Matrix Method for Mode Conversion at a Point Defect

To quantitatively unravel the underlying physics, we developed a model based on the scattering matrix method. The total electric field on the NW is expressed as a superposition of the eigenmodes:

(1) $${\vec{E}}_{\text{total}}\left(x,y,z\right)=\sum jbj\left(z\right)\vec{E}j\left(x,y\right)$$

where ${\vec{E}}_{j}\left(x,y\right)$ is the normalized field profile of the j‐th eigenmode (TM_0_, HE_−1_, HE_1_) and $b_{j}\left(z\right)$ is its complex amplitude coefficient. Propagation along a defect‐free segment over a distance Δz introduces a phase shift: $b_{j}\left(z+\Delta z\right)=b_{j}\left(z\right)e^{ik_{zj}\Delta z}$, where $k_{zj}=\frac{2\pi }{\lambda_{0}}$, $n_{\text{eff},j}$ is the effective refractive index of the modes, and $\lambda_{0}$ is the wavelength in free space.

The nanogroove is treated as a localized scatterer, coupling the incident and transmitted modal amplitudes via a 3 × 3 transmission matrix *T*:

(2) $$\left[\begin{array}{c} {b_{1}}^{\prime }\\ {b_{2}}^{\prime }\\ {b_{3}}^{\prime } \end{array}\right]=\left[\begin{array}{ccc} T11 & T12 & T13\\ T21 & T22 & T23\\ T31 & T32 & T33 \end{array}\right]\left[\begin{array}{c} b1\\ b2\\ b3 \end{array}\right]$$

The matrix elements $T_{\text{mn}}$ were extracted from dedicated FDTD simulations by using each eigenmode as an independent source and performing mode decomposition before and after the defect (see Methods).

The propagation and conversion characteristics of the SPP modes are quantified by the modal power $P_{j}\left(z\right)={\left|a_{j}\left(z\right)\right|}^{2}\cdot N_{j}$, where $a_{j}\left(z\right)$ is the complex amplitude and $N_{j}$ is the normalization factor (see Methods, Eigenmode Analysis). The SMM framework reliably reproduces full‐wave FDTD results (Supporting Information S1: Figure S2), validating its accuracy in capturing the system's electromagnetic response. It enables quantitative analysis of defect‐induced mode conversion and amplitude/phase modulation of each pathway. Moreover, systematic scanning of geometric parameters via SMM efficiently identifies the optimal dimensions that maximize the chiral extinction ratio $\eta =\frac{P_{+45^{\circ }}}{P_{-45^{\circ }}}$ under ± 45° excitation, a capability we exploit in the following section.

### 2.3 Optimization of the Nanogroove Geometry

The chiral extinction ratio depends critically on the nanogroove dimensions, which govern the amplitudes and phases in the T‐matrix. A single rectangular nanogroove is centered at the position where the near‐field intensity of SPP modes reaches its maximum, that is at a distance of 1.75 μm from the excitation end, consistent with the position adopted in Figure 1/the FDTD Simulation case in Section 2.1. The core design principle for high‐performance chiral filtering is to maximize the transmission contrast (extinction ratio) between SPPs of opposite chiralities. The optimal groove dimensions can be screened via geometric parameter sweeping; with the optimized parameters, the structure effectively suppresses the transmission of the undesired chirality and achieves the maximum transmission contrast under ± 45° polarization excitation. We systematically investigated this dependence by varying the depth $L_{y}$ (Figure 2) and width $L_{z}$ (Figure 3).

**FIGURE 2:**
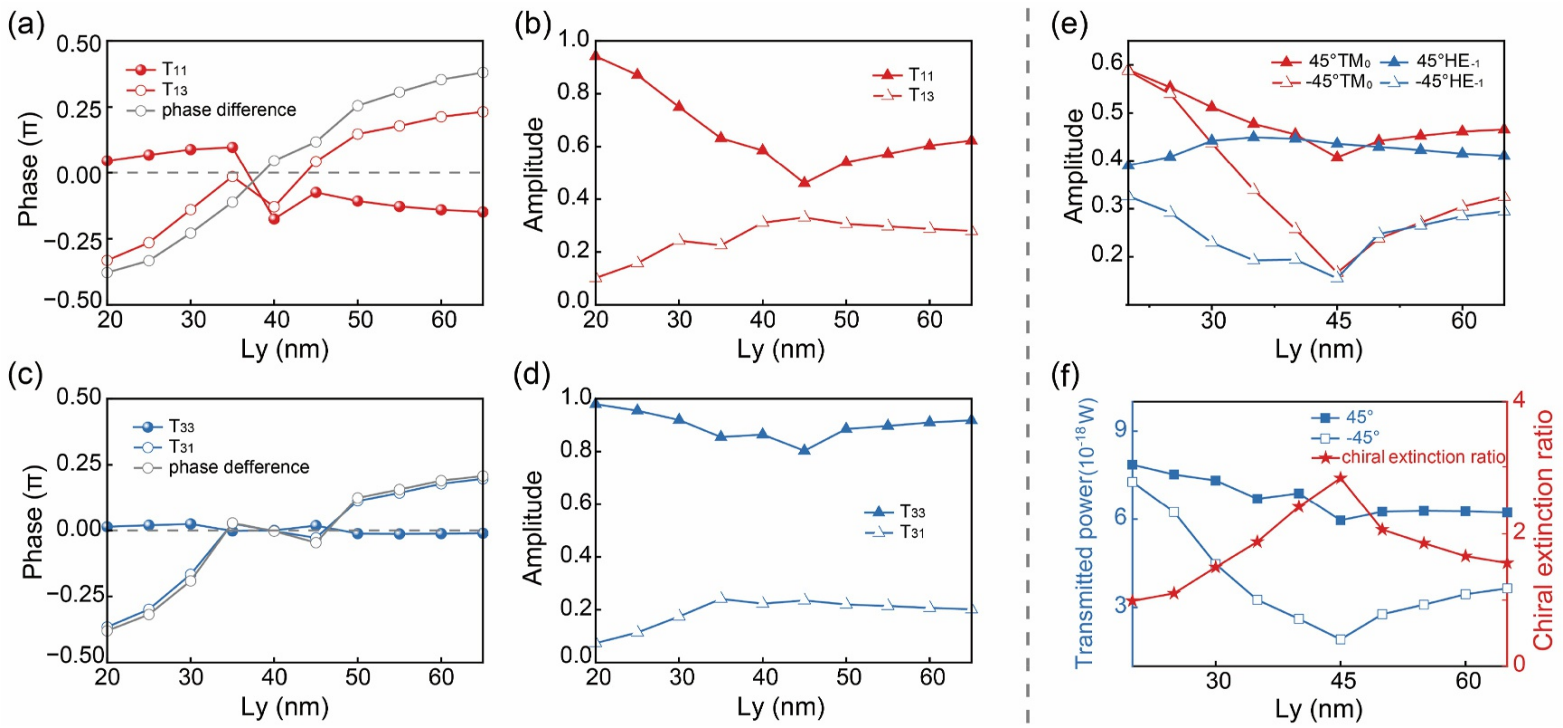
Dependence of the scattering properties and chiral filtering performance on nanogroove depth ($L_{y}$). (a, b) Phase and amplitude of the transmission matrix elements $T_{11}$ (TM_0_→TM_0_) and $T_{13}$ (HE_−1_→TM_0_). (c, d) Phase and amplitude of $T_{33}$ (HE_−1_→HE_−1_) and $T_{31}$ (TM_0_→ HE_−1_). The gray line in (a) and (c) indicates the phase difference between the two components. (e) Composite amplitude of the TM_0_ and HE_−1_ modes after the defect under ± 45° polarized excitation. (f) Total transmitted power and the chiral extinction ratio (η, red line) as a function of $L_{y}$. The width of the nanogroove is fixed as $L_{z}=55\;\text{nm}$.

**FIGURE 3:**
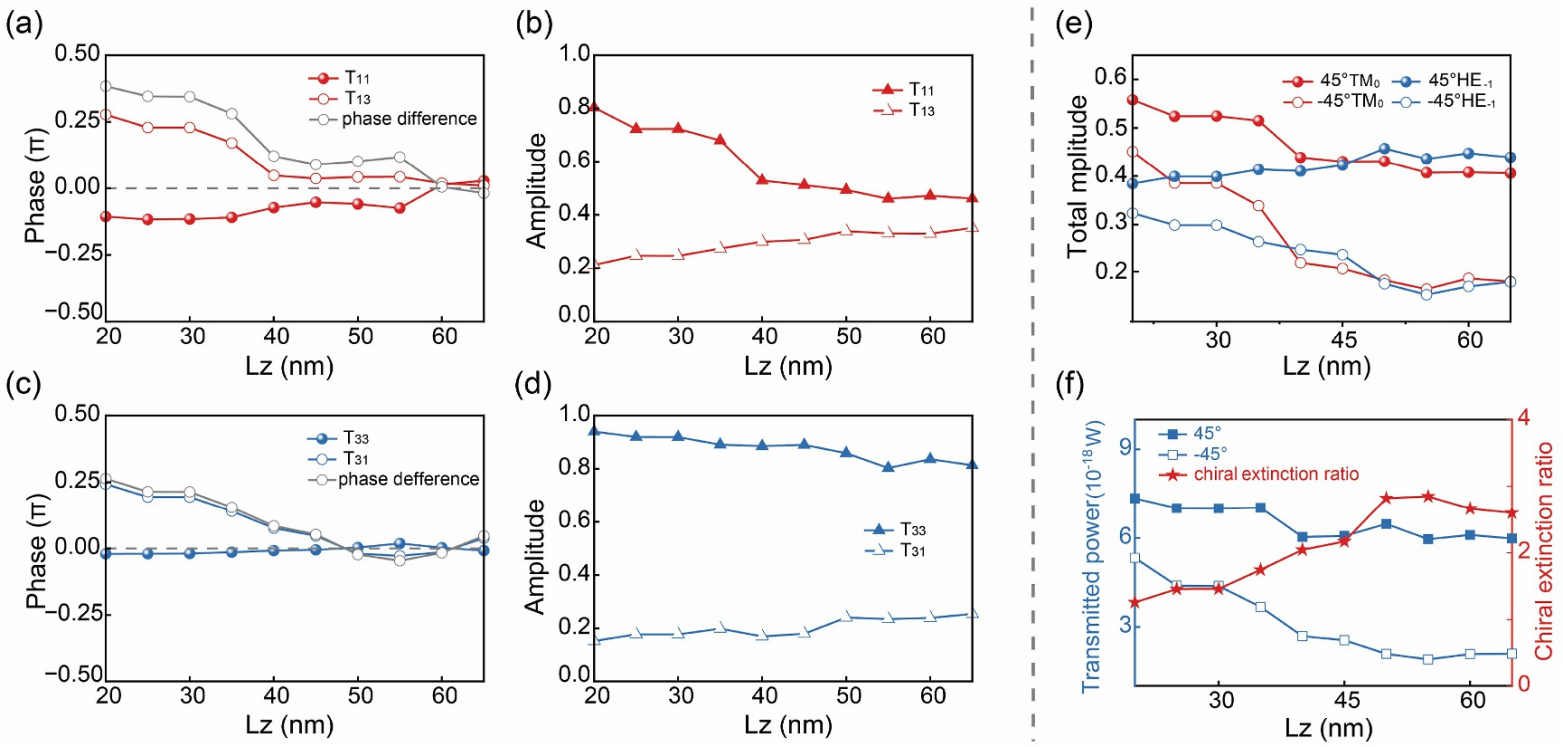
Dependence of the scattering properties and chiral filtering performance on nanogroove width ($L_{z}$). (a, b) Phase and amplitude of the transmission matrix elements $T_{11}$ (TM_0_→ TM_0_) and $T_{13}$ (HE_−1_→TM_0_). (c, d) Phase and amplitude of $T_{33}$ (HE_−1_→HE_−1_) and $T_{31}$ (TM_0_→HE_−1_). The gray line in (a) and (c) indicates the phase difference. (e) Composite amplitude of the TM_0_ and HE_−1_ modes after the defect under ± 45° polarized excitation. (f) Total transmitted power and the chiral extinction ratio (η, red line) as a function of $L_{z}$. The depth of the nanogroove is fixed as $L_{y}=45\;\text{nm}$.

As $L_{y}$ increases from 20 to 65 nm, the amplitudes of $T_{11}$ (TM_0_→ TM_0_) and $T_{13}$ ​(HE_−1_→TM_0_) exhibit resonant‐like but opposite trends, whereas their phase difference varies monotonically (Figure 2a,b). The trend reversal in amplitudes near $L_{y}$ = 45 nm signifies a resonant scattering condition. At this depth, the direct and converted components for the TM_0_ mode under −45° excitation are out‐of‐phase with comparable magnitudes, leading to a minimum in the composite TM_0_ amplitude (Figure 2e, hollow red triangles) due to destructive interference. This results in a minimum total transmitted power contribution for −45° excitation (Figure 2f). Conversely, for +45° excitation, the interference is constructive, and the transmitted power is less sensitive to $L_{y}$. Consequently, the ratio η peaks at $L_{y}$ = 45 nm. A parallel analysis of the elements $T_{33}$ (HE_−1_→HE_−1_) and $T_{31}$ (TM_0_→HE_−1_) (Figure 2c,d) shows a similar resonant behavior for the HE_−1_ mode, further contributing to the overall chiral filtering effect.

A similar analysis for width variation $L_{z}$ (Figure 3) shows that increasing $L_{z}$ primarily attenuates the direct transmission amplitude $\left|T_{11}\right|$ and $\left|T_{33}\right|$, while enhancing the mode conversion strength $\left|T_{13}\right|$ and $\left|T_{31}\right|$. The phase differences between the key matrix elements approach zero in the range of $L_{z}$ = 50–60 nm (Figure 3a,c). This geometric adjustment fine‐tunes the interference condition. The optimal balance for achieving the maximum contrast in the composite amplitudes of both TM_0_ and HE_−1_ modes for ± 45° excitation (Figure 3e), and thus the maximum η, is found at $L_{z}$ = 55 nm (Figure 3f). As $L_{z}$​ increases in Figure 3f, the transmitted power for +45° excitation exhibits an overall downward trend, with a sharp drop between 35 and 40 nm followed by an anomalous recovery from 40 to 50 nm. For −45° excitation, the transmitted power decreases more significantly over the range of 20–55 nm and then stabilizes for $L_{z}$ > 55 nm. These distinct responses of the transmitted powers for the two polarizations to variations in $L_{z}$​ lead to a maximum chiral extinction ratio at $L_{z}$ = 50–55 nm, followed by a gradual decline as $L_{z}$ increases further (Supporting Information S1: Figure S3).

The simultaneous optimization of both dimensions ($L_{y}$ = 45 nm, $L_{z}$ = 55 nm) yields the maximum theoretical η of 2.77 for a single groove, by establishing a configuration where interference and amplitude variation are both optimized. The contrasting behaviors of the transmitted powers for ± 45° excitations as functions of $L_{y}$ and $L_{z}$ highlight the complex yet selective influence of the nanogroove defect on chiral plasmonic signals.

For an optimized nanogroove (depth $L_{y}$ = 45 nm, width $L_{z}$ = 55 nm) at the specified position, the obtained transmission matrix *T* is:

(3) $$\left[\begin{array}{ccc} 0.492e^{0.026\pi i} & 0 & 0.305e^{0.013\pi i}\\ 0 & 0.996e^{0.013\pi i} & 0\\ 0.22e^{-0.021\pi i} & 0 & 0.87e^{0.003\pi i} \end{array}\right]$$

This matrix reveals key insights: (i) The near‐unity $T_{22}$ confirms the defect has negligible effect on the HE_1_ mode. (ii) Significant off‐diagonal components $T_{13}$ and $T_{31}$ indicate strong mutual conversion between the TM_0_ and HE_−1_ modes. (iii) The diagonal elements $T_{11}$ and $T_{33}$, as well as the conversion elements, introduce only minor phase shifts, confirming that the defect's primary role is to enable mode conversion without drastically altering the phase of individual components.

Using this transmission matrix *T* and the propagation phases, the calculated power evolution (Figure 4) reproduces the FDTD results, yielding a chiral extinction ratio $\eta =\frac{P_{+45{}^{\circ}}}{P_{-45{}^{\circ}}}$ of 2.77 for a single defect. The stark difference in transmitted power for ± 45° excitations originates from the distinct initial phase relationship between the TM_0_ and HE_−1_ modes upon reaching the defect. For +45° excitation, the two modes arrive nearly in‐phase, whereas for −45° excitation, they arrive nearly out‐of‐phase. After interacting with the defect, the directly transmitted component of a given mode (e.g., TM_0_ via $T_{11}$) interferes with the component converted from the other mode (e.g., TM_0_ via $T_{13}$ from HE_−1_). For +45° excitation, this interference is constructive because the total phase difference remains near 0. For −45° excitation, it becomes destructive as the total phase difference approaches π. This interference mechanism selectively suppresses or enhances the transmission for opposite chiralities, forming the physical basis of the filter's operation.

**FIGURE 4:**
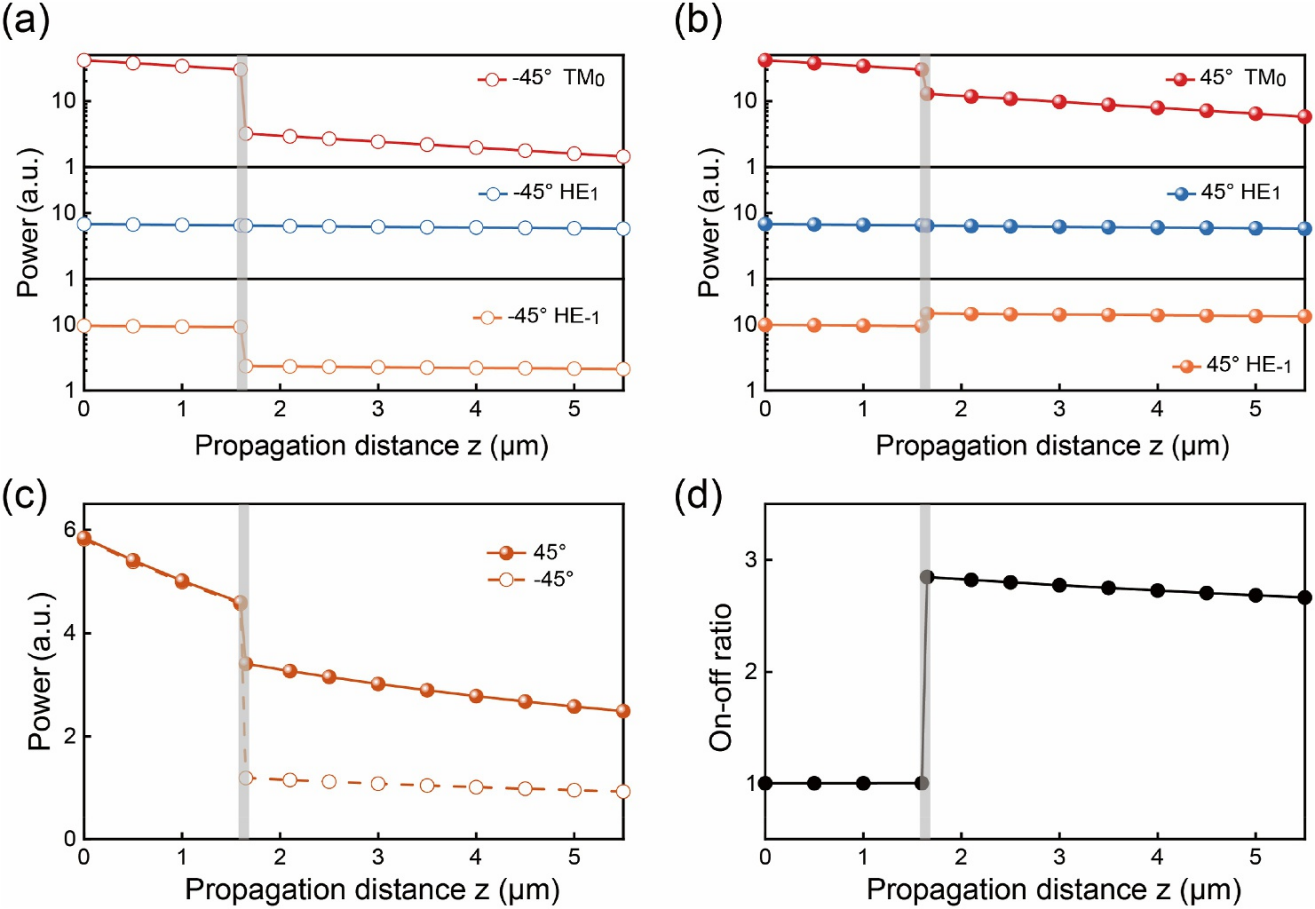
Mode‐resolved and total power evolution calculated via the scattering matrix method. (a, b) Propagating power in the TM_0_, HE_1_, and HE_−1_ modes as a function of distance under −45° (a) and +45° (b) polarized excitation. The gray shaded region indicates the location of the nanogroove. (c) Evolution of the total transmitted power for both excitations. (d) The corresponding chiral extinction ratio, $\eta =P_{+45{}^{\circ}}/P_{-45{}^{\circ}}$, as a function of propagation distance. The dimensions of the nanogroove are set as $L_{y}=45\;\text{nm}$ and $L_{z}=55\;\text{nm}$.

### 2.4 Influence of Defect Number (N) and Spacing

The scattering matrix method readily extends to multiple defects. The total transmission for identical grooves separated by a distance Δz is described by cascading the defect matrix *T* and propagation matrices P(Δz): $b^{\prime }={\left(TP\right)}^{N}b$. A comparison with the full cascaded model accounting for multiple reflections is provided in Supporting Information S1: Figure S5.

First, fixing defect number *N* = 3, we calculated the transmitted power as a function of Δz (Figure 5a). Both transmitted power $P_{+45{}^{\circ}}$ and $P_{-45{}^{\circ}}$ oscillate due to Fabry–Pérot‐like interference between defects, but with opposite phases. The chiral extinction ratio η reaches a maximum of 3.69 at an optimal spacing of Δz = 1.75 μm, which closely matches both the beat length for a cylindrical wire ($L_{beat}$ = 1.81 μm, from $L_{beat}$ = *λ*_0_/[$n_{\text{eff}}\left({\text{TM}}_{0}\right)$ − $n_{\text{eff}}\left({\text{HE}}_{-1}\right)$]) and the measured value ($L_{beat}$ = 1.83 μm, Supporting Information S1: Figure S4). The measured value is approached by a sequence of increasingly realistic cross‐sectional models (Supporting Information S1: Table S1): a bare pentagonal cross‐section gives 1.47 μm; adding a 10‐nm Al_2_O_3_ shell raises this to 1.65 μm; and the cylindrical model gives 1.81 μm. The monotonic progression from 1.47 μm toward the experimental value of 1.83 μm demonstrates that the shell and the corner rounding, both present in the real nanowire, systematically shift the modal dispersion in the direction of the measured beat length. The residual difference is attributed to the geometric variability of chemically synthesized AgNWs and to local dielectric inhomogeneity.

**FIGURE 5:**
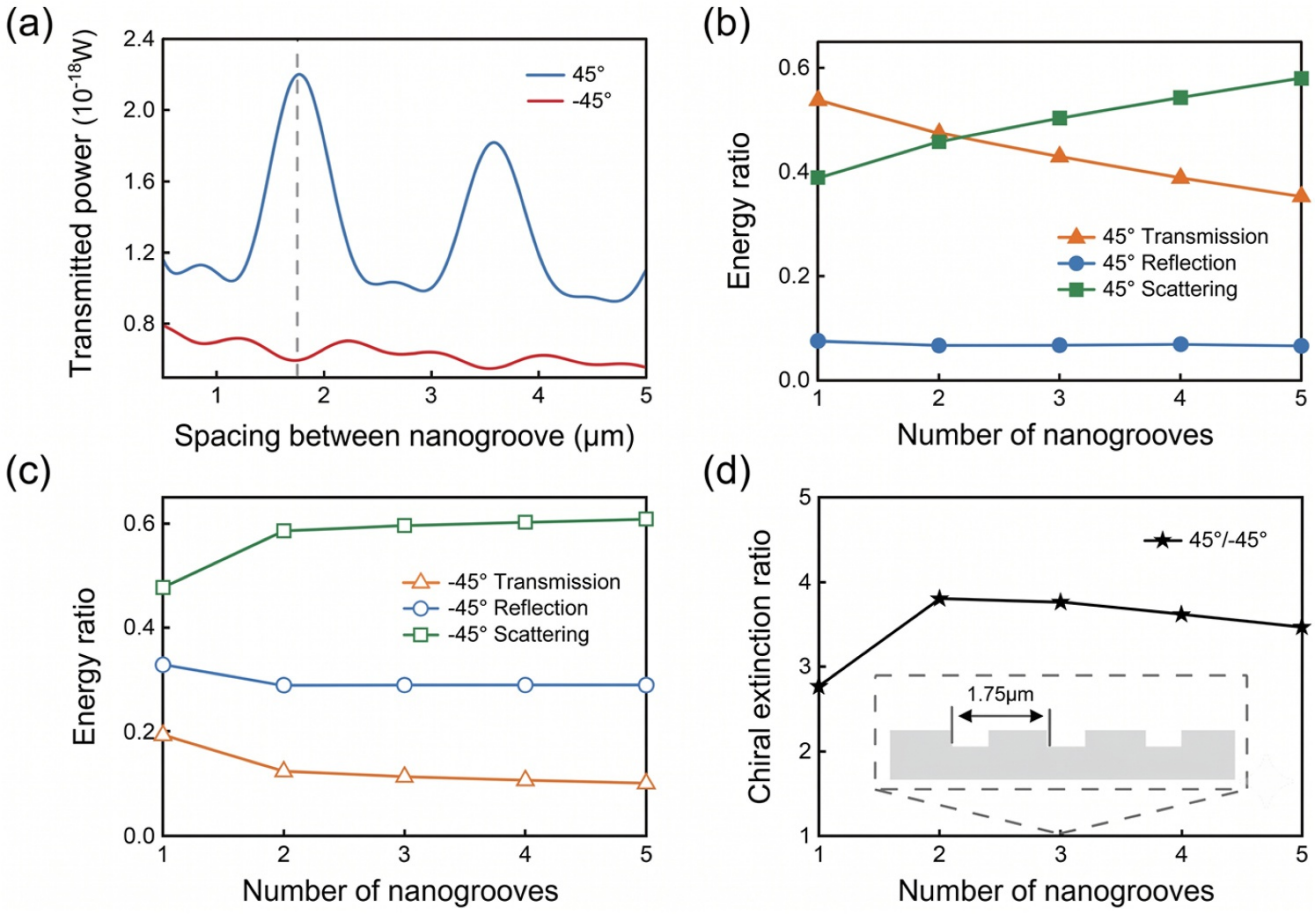
Performance of chiral filters with multiple nanogrooves. (a) Transmitted power for ± 45° polarized excitation as a function of the spacing (Δz) between three identical nanogrooves. The maximum chiral extinction ratio (*η*) occurs at Δz = 1.75 μm (gray dashed line). (b, c) Transmittance, reflectance, and scattering efficiency versus the number of nanogrooves (N) under +45° (b) and −45° (c) polarized excitation, with spacing fixed at 1.75 μm. (d) Dependence of the chiral extinction ratio *η* on the number of grooves (N). For each nanogroove, $L_{y}$ = 45 nm, $L_{z}$ = 55 nm. The first nanogroove's center is placed 1.75 μm from the excitation end. For (b–d), the nanogroove center‐to‐center spacing is 1.75 μm.

Second, fixing Δz at this optimal value, we increased the number of defects *N* (Figure 5b–d). For +45° excitation, transmittance decreases gradually while reflectance remains low. For −45° excitation, transmittance drops sharply from *N* = 1 to *N* = 2, after which it decreases more slowly, accompanied by a higher reflectance. The chiral extinction ratio η peaks at *N* = 2, reaching a value of ∼3.7 (consistent with the full cascaded model, Supporting Information S1: Figure S5), demonstrating the potential for enhanced performance with simple multi‐defect designs.

### 2.5 Experimental Demonstration of Chiral Plasmonic Filtering

To validate our theoretical predictions, we fabricated a chiral filter on a monocrystalline Ag NW (diameter ∼120 nm, length 8.9 μm) using focused ion beam (FIB) milling. A single V‐shaped nanogroove (width ∼83 nm, depth ∼45 nm) was etched at a distance of 4.86 μm from one end (Figure 6a) to prevent the laser spot illuminating the defect. This location is close to the third near‐field fluorescence maximum under ± 45° polarized excitation. The constraint of avoiding the 2–3 μm excitation spot prevents placing the groove at the first two maxima. The sample was then coated with a 10 nm Al_2_O_3_ layer via atomic layer deposition (ALD) and spin‐coated with CdSe/ZnS quantum dots (QDs, emission ∼650 nm) for near‐field visualization. Subsequently, the sample was immersed in oil with a refractive index of 1.5.

**FIGURE 6:**
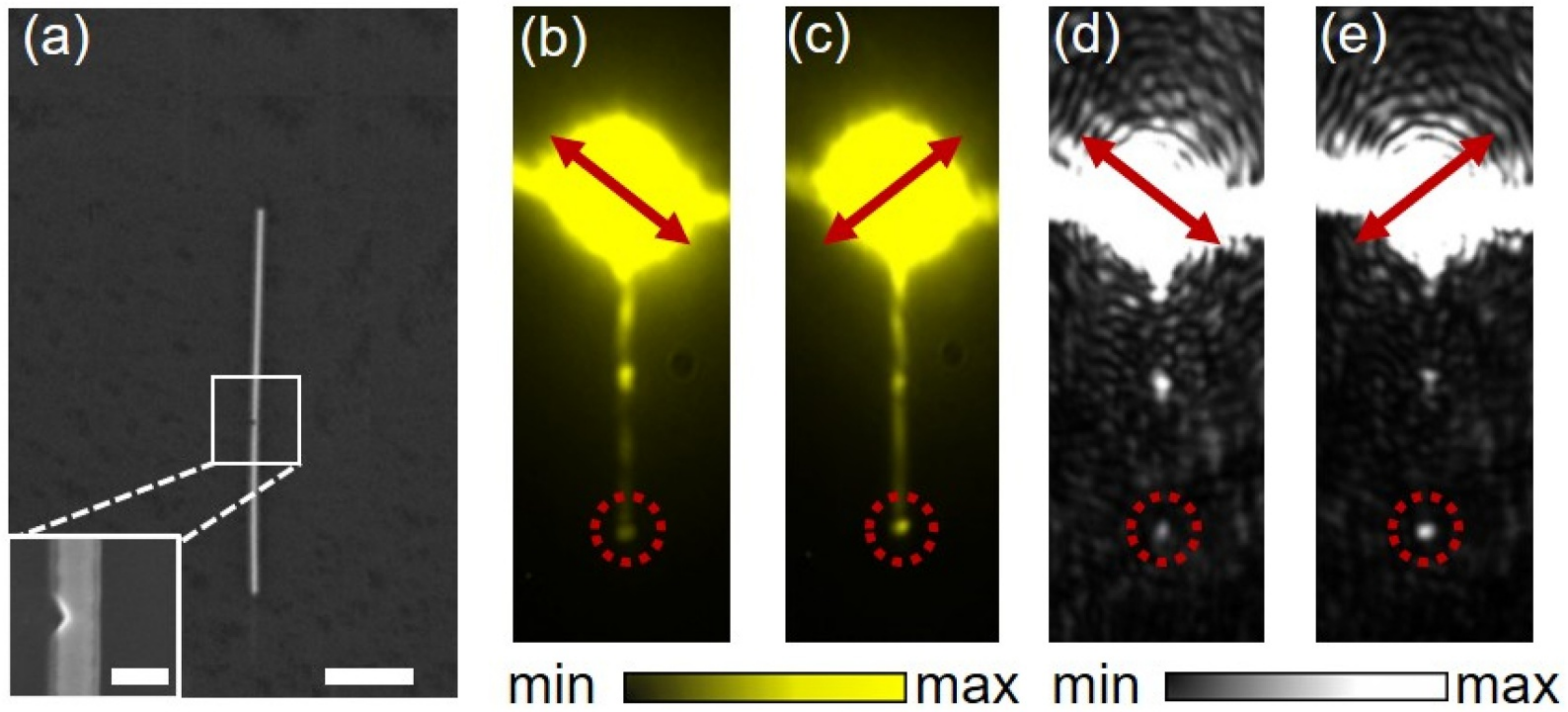
Experimental demonstration of chiral plasmonic filtering. (a) Scanning electron microscope (SEM) image of the fabricated Ag NW (diameter ∼120 nm, length 8.9 μm) with a single V‐shaped nanogroove (width ∼83 nm, depth ∼45 nm). Scale bar: 2 μm. Inset: Magnified view of the nanogroove, whose center is positioned 4.86 μm from the excitation end. Scale bar: 200 nm. (b, c) Fluorescence images from quantum dots coated on the NW under −45° (b) and +45° (c) polarized excitation, revealing the near‐field distribution of the chiral SPPs. (d, e) Corresponding far‐field optical scattering images from the NW termination under −45° (d) and +45° (e) polarization excitation. The red arrows indicate the polarization direction of the excitation laser.

Under ± 45° polarized 633 nm laser excitation, the chiral SPPs were launched and propagated along the NW. Their near‐field profiles were imaged via QD fluorescence (Figure 6b,c), showing the characteristic spiral intensity patterns with opposite handedness. The far‐field scattering from the NW termination was also recorded (Figure 6d,e). A clear intensity contrast is observed at the remote end: the scattering signal for +45° excitation is significantly stronger than that for −45° excitation. Quantitatively, the ratio of the end‐scattering intensities yields an experimental chiral extinction ratio of 1.96 (Supporting Information S1: Figure S6). To place this value in context, we estimated the expected extinction ratio for the actual fabricated geometry. An 83‐nm‐wide rectangular groove (matching the nominal width of the V‐shaped notch) would yield *η* ≈ 2.5 in simulation; when the smaller average depth of the V‐shaped profile is taken into account, this estimate is reduced to *η* ≈ 2.0–2.2. The measured value of 1.96 is therefore dominated by the well‐understood geometric effect. The fluorescence images (Figure 6b,c) and far‐field scattering images (Figure 6d,e) provide complementary information. At the nanogroove position, the fluorescence intensity for −45° excitation is approximately 2.7 times that for +45° excitation, reflecting the local field enhancement at the defect where −45° excitation produces constructive interference. By contrast, the transmitted power at the distal end, which integrates the modal energy over the nanowire cross‐section, exhibits an opposite trend. This apparent inversion is not contradictory but arises from the distinct physical quantities being probed: fluorescence images the local near‐field intensity, which is sensitive to the field maximum at the defect, whereas scattering images measure the integrated power that survives propagation past the defect. Additionally, the QD fluorescence signal is subject to saturation and polarization‐dependent excitation efficiency, which may further modify the apparent contrast. The two observables therefore provide complementary validation of the chiral filtering mechanism.

Several primary factors account for the quantitative mismatch between numerical predictions and experimental observations, as detailed below. First, although the cylindrical approximation captures the core physics, the intrinsic pentagonal cross‐section of chemically synthesized AgNWs introduces minor shifts in the mode effective indices, leading to subtle phase deviations over the propagation length. Second, the actually fabricated notches exhibit a V‐shaped profile, which differs from the ideal rectangular model. Furthermore, maintaining a low ion dose during alignment to prevent Ga^+^ implantation damage introduces a trade‐off in positional accuracy. Nevertheless, the consistent experimental observation of the predicted chiral intensity inversion and spiral near‐field patterns underscores the robustness of the proposed physical mechanism under realistic environmental constraints.

The groove position is a genuine design parameter of the device. To quantify its role, we calculated *η* for a single groove as a function of its position $z_{0}$ using the SMM (Supporting Information S1: Figure S11). The calculation shows that *η* oscillates with position, with local maxima spaced by 1.811 μm—in excellent agreement with the theoretical beat length—and with the peak value decaying monotonically with distance due to the propagation loss. The first maximum occurs at $z_{0}\;\approx \;1.68\;\mu m$, which is very close to the value used in our FDTD simulations (1.75 μm). The experimentally chosen position (4.86 μm) lies about 0.44 μm away from the third maximum (5.30 μm). The difference is attributed to the V‐shaped groove profile and to the possible shift of the fluorescence maximum.

## 3 Conclusions

In summary, we have demonstrated that a single nanogroove, acting as a deterministically engineered point defect, can function as an effective chiral filter for surface plasmon polaritons propagating on silver nanowires. This work introduces nanoscale defect engineering as a compact and powerful regulatory dimension for on‐chip plasmonic control, moving beyond conventional reliance on excitation polarization alone. Through integrated FDTD simulations and scattering matrix method, we quantitatively revealed that the filtering mechanism stems from defect‐induced selective mode conversion (primarily between TM_0_ and HE_−1_ modes) and the subsequent coherent interference between direct transmission and mode‐converted pathways.

By optimizing the nanogroove geometry (depth: 45 nm, width: 55 nm), we achieved a simulated chiral extinction ratio η of 2.77. Experimental verification for the single‐groove device, employing FIB‐fabricated nanowires and characterized via both far‐field scattering and quantum‐dot fluorescence imaging, yielded a measured η of 1.96, confirming the practical feasibility and validating our theoretical models. The SMM also predicts an enhanced *η* of ∼3.7 for two optimally spaced grooves, offering a clear route for future experimental exploration.

This study demonstrates a simple, localized strategy for manipulating chiral plasmonic propagation within individual nanowires, compatible with established nanofabrication techniques and suitable for developing ultra‐compact polarizers, beam splitters, and routers. The accompanying scattering matrix method offers a general, scalable framework for analyzing and designing plasmonic circuits with engineered defects by cascading basic units, effectively avoiding computationally expensive full‐wave simulations while linking intuitive physical insight with quantitative performance prediction. Thus, this defect‐oriented approach establishes a versatile pathway toward advanced integrated nanophotonic devices for optical information processing and sensing.

## 4 Methods

Numerical Simulations (FDTD): All three‐dimensional electromagnetic simulations were performed using a commercial FDTD solver (Ansys Lumerical). The simulation domain boundaries were set as perfectly matched layers (PML) in all directions. Ag NWs with a diameter of 120 nm was placed in a homogeneous dielectric background with a refractive index of *n* = 1.5, matching the index‐matching oil used in experiments. The complex refractive index of silver was taken from the experimental data of Johnson and Christy (*λ* = 632.8 nm: *n* = 0.0559 + 4.28i) [36].

Eigenmode Analysis: The eigenmodes of the pristine Ag NWs were calculated using the built‐in mode analysis tool. A mode source, set at a wavelength of 632.8 nm, was placed at the input end of the NW to excite SPPs. The effective refractive indices for the three supported fundamental modes were found to be: $n_{\text{eff}}\left({\text{TM}}_{0}\right)=1.90+0.01i$, $n_{\text{eff}}\left({\text{HE}}_{\pm 1}\right)=1.55+0.00134i$. The power of the TM_0_ and HE _±_ _1_ modes with unit amplitude are 1.334 × 10^−17^ W and 1.330 × 10^−17^ W, respectively. Although actual AgNWs exhibit pentagonal cross‐sections [37], our numerical analysis confirms that the fundamental mode symmetries and their degeneracy—crucial for chiral propagation—are well‐preserved. The slight variation in the effective index of the TM_0_ mode (*n*_eff_ ≈ 1.98 for pentagonal vs. 1.90 for cylindrical, see Supporting Information S1: Table S1 for a systematic comparison) primarily scales the beat length without changing the underlying mode conversion and interference principles.

Extraction of the Scattering Matrix for a Point Defect: To obtain the transmission (T) and reflection (R) matrices of a single nanogroove, we performed a set of dedicated FDTD simulations. Each of the three eigenmodes (TM_0_, HE_1_, HE_−1_) was independently launched as a mode source from one side of NWs containing a single rectangular nanogroove. Two mode expansion monitors were placed before and after the defect. By recording the complex amplitude coefficients for all three modes at each monitor, the forward transmission ($T_{\text{mn}}$) and reflection ($R_{\text{mn}}$) coefficients from an incident mode *n* to a scattered mode *m* were directly calculated. This process yields the full 3 × 3 T and R matrices characterizing the point defect.

Simulation of Chiral Excitation and Field Profiles: To simulate the chiral filtering phenomenon, the excitation source was switched to a linearly polarized Gaussian beam (wavelength 632.8 nm) focused onto the NWs end‐face. The polarization angle was set to ± 45° relative to the nanowire axis to generate chiral SPPs. Field profiles, including cross‐sectional and unfolded azimuthal views, were recorded to visualize the propagation and filtering effect.

Theoretical Framework: Cascaded Scattering Matrix Method: The system with multiple defects was analyzed using a cascaded scattering matrix method. The scattering matrix $S_{i}$ for the i‐th defect (or a propagation segment) is defined as:

(4) $$Si=\left[\begin{array}{cc} Ri & T_{i}^{p}\\ Ti & Ri^{p} \end{array}\right]$$

where $R_{i}$ and $T_{i}$ are the reflection and transmission matrices for waves incident from the left, and $R_{i}^{p}$ and $T_{i}^{p}$ are those for waves incident from the right. The total scattering matrix $S_{total}$ for a cascade of components (e.g., defect‐spacing‐defect) is calculated using the Redheffer star product, which correctly accounts for all multiple reflections and interference between components [38]. For the analysis in Sections 2.4, the propagation matrix $P\left(\Delta z\right)=\text{diag}\left(\exp\left(ik_{zj}\Delta z\right)\right)$ for a spacing Δz was integrated into this cascade formalism.

Nanowire Synthesis and Sample Preparation: Silver nanowires were synthesized via a modified polyol process and dispersed in ethanol [39]. To facilitate positioning and subsequent alignment, metallic alignment marks were prefabricated on quartz substrates. Quartz substrates were sequentially cleaned ultrasonically with acetone, ethanol, and deionized water, followed by drying under high‐purity N_2_. A layer of S1813 positive photoresist was spin‐coated at 4000 rpm for 60 s, and the alignment patterns were defined using a UV lithography system with a 25 s exposure. Following development, a 50‐nm‐thick Au film was deposited via thermal evaporation, and a standard lift‐off process in acetone yielded the final positioning marks. A 10 μL droplet of the purified AgNW solution (ultrasonicated for 10 s to ensure dispersion) was diluted with 90 μL of ethanol and cast onto the patterned substrate, allowing the nanowires to settle naturally under gravity before rinsing and nitrogen blow‐drying.

Focused Ion Beam (FIB) Milling of Nanogrooves: Individual AgNWs were located under a focused ion beam scanning electron microscope (FIB‐SEM, TESCAN SOLARIS) operating at an acceleration voltage of 30 kV. To suppress charge accumulation and eliminate ion beam drift during milling, a thin layer of water‐soluble conductive adhesive was spin‐coated (3000 rpm, 60 s) onto the substrate. To minimize lattice damage and Ga^+^ ion implantation, nanowires were identified using a single, rapid scan over a large field of view (300 × 300 μm^2^). The beam current (2.2 × 10^−4^ pC/μm^2^) for ion beam positioning and observation were strictly limited to minimize Ag NWs damage by Ga^+^ implantation. Nanogrooves were subsequently milled using optimized beam current of 10 pA and an aperture of 40 μm for a dwell time of 2 s.

Atomic Layer Deposition (ALD) and Quantum Dot Coating: A 10‐nm‐thick Al_2_O_3_ layer was conformally deposited over the entire sample at 150°C using thermal ALD (MNT, model ALS100E) with trimethylaluminum and H_2_O as precursors. This layer serves to prevent oxidation of Ag and to mitigate fluorescence quenching of the subsequent quantum dots (QDs). Finally, a toluene solution of CdSe/ZnS core‐shell QDs (QDSOL‐650, Wuhan Jiayuan Quantum Dot Co., emission peak ∼650 nm) was spin‐coated onto the substrate at 2000 rpm for 30 s. The uniform distribution of the QDs and the conformal nature of the Al_2_O_3_ layer on the AgNWs were unequivocally verified via energy‐dispersive x‐ray spectroscopy (EDS) mapping (Supporting Information S1: Figure S7).

Optical Characterization: Optical measurements were performed on an inverted microscope (Olympus BX51). A 633 nm He‐Ne laser was passed through a linear polarizer and a half‐wave plate to control the excitation polarization. The beam was focused onto one end of the target Ag NW using an oil‐immersion objective (100 ×, NA 1.4, Olympus). To ensure a symmetric dielectric environment—which is essential for the optimal excitation of chiral SPPs—a refractive index matching oil was applied over the sample. For far‐field scattering imaging, the light scattered from the NW (primarily at the remote termination) was collected by the same objective and imaged onto a CCD camera (DVC 710M‐00‐MW). For fluorescence imaging, a long‐pass filter (cut‐on wavelength 645 nm) was inserted in the collection path to block the laser light and transmit only the QD photoluminescence, thereby mapping the near‐field intensity of the propagating SPPs.

## Author Contributions

**Huijie Wang:** writing – original draft, methodology, data curation, software, investigation, formal analysis, visualization, writing – review and editing. **Yifan Chen:** software, writing – review and editing. **Wei Dai:** writing – review and editing. **Wenjun Zhang:** conceptualization, writing – review and editing. **Wenqiang Wang:** software, writing – review and editing. **Li Chen:** formal analysis, writing – original draft, writing – review and editing. **Zhiqiang Guan:** conceptualization, supervision, funding acquisition, validation, writing – review and editing, formal analysis, project administration, resources, writing – original draft. **Hongxing Xu:** supervision, funding acquisition, writing – review and editing, conceptualization, project administration, resources, writing – original draft.

## Funding

The work was supported by National Natural Science Foundation of China (Grants 52488301 and 12574338), Natural Science Foundation of Henan Province (No. 252300421055), and Fundamental Research Funds for the Central Universities (No. 2042022kf0047).

## Conflicts of Interest

The authors declare no conflicts of interest.

## Supporting information


Supporting Information S1

## Data Availability

The data that support the findings of this study are available from the corresponding author upon reasonable request.
