# Supplementary material for: Chiral Plasmonic Filtering in Silver Nanowires via Point‐Defect‐Induced Mode Conversion and Interference
Source: Nanophotonics. 2026 Nov 5;15(21):e70324. doi: 10.1002/nap2.70324 (PMC13735899; doi:10.1002/nap2.70324)
Supplement: Supplementary file 1 [file NAP2-15-e70324-s001.docx]

Supporting Information for

**Chiral Plasmonic Filtering in Silver Nanowires via Point-Defect-Induced Mode Conversion and Interference**

Huijie Wang^1^, Yifan Chen^1^, Wei Dai^1^, Wenjun Zhang^2^, Wenqiang Wang^2^, Li Chen^3^, Zhiqiang Guan^1, 4, *^, Hongxing Xu^4, *^

^1^School of Physics and Technology, Center for Nanoscience and Nanotechnology, and Key Laboratory of Artificial Micro and Nanostructures of Ministry of Education, Wuhan University, Wuhan 430072, China

^2^Institute of Physics, Henan Academy of Sciences, Zhengzhou 450046, China

^3^Institute of Light Resources and Environmental Science, Henan Academy of Sciences, Zhengzhou 450046, China

^4^Henan Academy of Sciences, Zhengzhou 450046, China

*** Corresponding authors**：[gzq@hnas.ac.cn](mailto:gzq@hnas.ac.cn), [hxxu@whu.edu.cn](mailto:hxxu@whu.edu.cn).

### S1. Electric field distribution and circular polarization degree with three grooves introduced.

**Figure S1. Circular polarization degree of the field transmitted past three nanogrooves.** (a) Cross-sectional electric field distribution on the silver nanowire after passing the nanogrooves under +45° polarization excitation. (b) Expanded view of field distribution at 10 nm outside the silver nanowire; (c) Circular polarization degree *C* and (d) circular polarization quality factor *f* calculated at 200 nm from the distal end of the nanowire. The nanogroove dimensions are $L_{y}=45 \mathrm{nm}$ (depth) and $L_{z}=55 \mathrm{nm}$ (width), positioned at 1.75 μm from the excitation end with center-center spacing 1.75 μm. *C* reaches approximately 0.8, providing quantitative evidence of the chiral nature of the transmitted SPPs.

## S2. Reconstruction of Electric Field Distribution Using the Scattering Matrix Method

To validate the accuracy and predictive power of the Scattering Matrix Method (SMM) developed in the main text, we used it to reconstruct the total electric field distribution following the nanogroove and compared the result with a direct full-wave finite-difference time-domain (FDTD) simulation.

The complex amplitude coefficients for the three fundamental modes (TM₀, HE₁, and HE₋₁) excited by +45° polarization polarized light were recorded at an input monitor. These coefficients, combined with the transmission matrix T of the single nanogroove, were used to calculate the post-defect modal amplitudes. The total electric field distribution was then reconstructed by superimposing the known field profiles of each eigenmode weighted by its calculated complex amplitude.

Figure S2 presents a direct comparison. Figure S2a shows the unfolded azimuthal electric field distribution calculated via the SMM. Figure S2b displays the field distribution obtained from the full 3D FDTD simulation under identical excitation conditions. The relative difference in electric field intensity between the two methods, plotted in Figure S2c, is within 18% across the region of interest. A similar comparison for -45° excitation yields a relative difference within the same 18% range (data not shown). This excellent agreement confirms that the semi-analytical SMM framework accurately captures the electromagnetic response of the system, reliably reproducing the results of full-wave numerical simulations.

**Figure S2. Validation of electric field reconstruction via the Scattering Matrix Method (SMM) under +45° polarized excitation.** (a) Unfolded azimuthal electric field (|E|) distribution calculated using the SMM. (b) Corresponding field distribution from a full 3D FDTD simulation. (c) Relative percentage difference in electric field intensity between the SMM result (a) and the FDTD result (b). The difference is within 18%, validating the accuracy of the SMM.

**S3. Total transmitted power and the chiral extinction ratio as a function of** $\boldsymbol{L}_{\boldsymbol{z}}$**.**

Figure S3. Total transmitted power and the chiral extinction ratio (η, red line) as a function of $\boldsymbol{L}_{\boldsymbol{z}}$. The horizontal axis extends to $L_{z}$ = 100 nm. The depth of the nanogroove is fixed as $L_{y}=45 \mathrm{nm}$.

**S4. Quantum-dot Fluorescence Intensity distribution along the axis of the silver nanowire.**

Figure S4. Measured fluorescence intensity profile along the Ag NW axis under +45° polarized excitation. The period of the oscillation corresponds to the beat length $L_{beat}\approx1.83 \mu m$.

### S5. Cascaded Scattering Matrix Method with Multiple Reflections

In the main text, the analysis of multiple nanogrooves initially neglected multiple reflections between defects for clarity, using the simplified cascade formula $T_{\text{total}}={(TP)}^{N}$. For completeness, we also performed calculations using the full cascaded scattering matrix method (Redheffer star product), which rigorously accounts for all multiple reflections and Fabry-Pérot interference within the array.

The performance of a three-defect (N = 3) array was analyzed. Figure S5a plots the total transmitted power for ±45° polarization excitation as a function of defect spacing ($\Delta z$), calculated with the full multi-reflection model. The trend and the position of the optimal spacing (1.75 μm) are nearly identical to those obtained from the simplified model (main text Figure 5a). The maximum chiral extinction ratio $\eta$ considering multiple reflections is 3.76, slightly higher than the value of 3.69 from the simplified model, indicating a minor constructive interference contribution from reflected waves.

Furthermore, Figures S5b and S5c show the transmittance, reflectance, and scattering efficiency versus the number of grooves (N) for ±45° polarization excitation at the optimal fixed spacing of 1.75 μm. Figure S5d plots the corresponding chiral extinction ratio $\eta$. These results are in close agreement with those from the simplified model (main text Figures 5b-d), confirming that while multiple reflections have a quantifiable effect, the primary filtering behavior and optimization trends are robustly captured by the simpler analytical cascade.

**Figure S5. Performance of multi-defect chiral filters calculated with the full cascaded scattering matrix method (including multiple reflections).** (a) Transmitted power for ±45° polarization excitation as a function of spacing ($\Delta z$) between three identical nanogrooves (N = 3). The gray dashed line marks the optimal spacing of 1.75 μm where the chiral extinction ratio $\eta$ is maximized. (b, c) Transmittance, reflectance, and scattering efficiency as a function of the number of grooves (N) under +45° (b) and -45° (c) polarization excitation, with spacing fixed at 1.75 μm. (d) Dependence of the chiral extinction ratio $\eta$ on the number of grooves (N). These results are consistent with the simplified model presented in the main text.

### S6. Scattering Intensity profiles at the End of Ag NW

**Figure S6. Scattering intensity profiles at the end of Ag NW.** The scattering intensity profiles at the end of Ag NW was acquired via DVCView 3.5 software under 45° and -45° polarization excitation conditions. The maximum scattering intensity at the nanowire end is ~225 a.u. under 45° polarization excitation, while it is ~115 a.u. under -45° polarization excitation. The intensity contrast between the two conditions is calculated as 225/115≈1.96.

**S7. Structural and elemental characterization of the AgNW-QD system.**

Figure S7. (a) Scanning electron microscopy (SEM) image of a representative silver nanowire (AgNW) on a silicon substrate. (b-e) Energy-dispersive X-ray spectroscopy (EDS) elemental mapping results for Si (substrate), Cd (indicating CdSe/ZnS QDs), O (from the substrate and Al_2_O_3_ coating), and Ag (the nanowire), respectively, confirming the uniform distribution of quantum dots and the presence of the protective Al_2_O_3_ layer.

**S8. Comparative characterization of distal scattering responses and near-field fluorescence symmetry of AgNW under orthogonal ±45° incident polarizations.**

Figure S8. (a) Comparison of the distal scattering response for AgNW under ±45° incident polarizations. (i) and (ii) are the quantum dot fluorescence images at each polarization, showing a symmetric near-field distribution. (b) Quantitative comparison of the distal scattering intensities. These values are obtained from a pristine nanowire and are not directly comparable in absolute magnitude to the scattering intensities from the notched nanowire shown in Figure S6.

**S9. Photoluminescence (PL) spectrum of the CdSe/ZnS quantum dots (QDs).**

Figure S9. Photoluminescence (PL) spectrum of the CdSe/ZnS quantum dots (QDs). The spectrum shows a prominent emission peak at approximately 650 nm.

**S10. High-magnification SEM image of the V-shaped nanogroove fabricated by FIB milling.**

Figure S10. High-magnification SEM image of the V-shaped nanogroove fabricated by FIB milling. The groove has a width of approximately 83 nm and a depth of approximately 45 nm. This is the same device as shown in Figure 6a of the main text.

**S11: Single-groove position dependence of η**

**Figure S11. Dependence of the chiral extinction ratio η on the position of a single nanogroove.** $\eta={P_{45^{\circ}}}/{P_{-45^{\circ}}}$ calculated by the SMM as a function of the single-groove position $z_{0}$ over three beat periods ($\text{L}_{\text{beat}}=1.81 \mu m$). The groove dimensions are fixed at $L_{y}=45 \mathrm{nm}$ and $L_{z}=55 \mathrm{nm}$. Green triangles mark the local maxima of η; the first occurs at $z_{0}\approx\text{1.68 }\text{μm}$ and their spacing is constant at 1.81 μm. The red spot marks the experimental groove position (4.86 μm).

**S12. Mode analysis of AgNW cross-sectional models and the beat length (Table S1).**

To reconcile the measured beat length with the actual cross-sectional geometry of the AgNW, we performed eigenmode calculations for four cross-sectional models of increasing realism. The effective indices of the TM₀ and HE₋₁ modes and the corresponding theoretical beat lengths are summarized in Table S1. The table shows a monotonic progression of $L_{\mathrm{beat}}$ from 1.47 μm (bare pentagonal) to 1.65 μm (pentagonal + 10-nm Al₂O₃ shell, with a conservative 2-nm corner rounding) and to 1.81 μm (cylindrical), approaching the experimental value of 1.83 μm. The cylindrical model is therefore best regarded as a convenient limiting case that numerically matches the experiment, rather than as a faithful description of the actual cross-section. The residual difference between the coated pentagonal model (1.65 μm) and the experiment (1.83 μm) is attributed to the inherent morphological variability of chemically synthesized AgNWs — in particular, larger and more irregular corner rounding than the conservative 2-nm estimate, and small deviations of the actual diameter from the ideal 120 nm used in simulation.

**Table S1. Effective indices and beat lengths for different cross-sectional models of the AgNW.**

| Model | $n_{\text{eff}}(\text{TM}_{0})$ | $n_{\text{eff}}(\mathrm{HE}_{-1})$ | $\Delta n$ | $L_{beat} (\mu m)$ |
| --- | --- | --- | --- | --- |
| Bare pentagonal | 1.98 | 1.55 | 0.43 | 1.47 |
| Pentagonal + corner rounding (2 nm) | 1.96 | 1.55 | 0.41 | 1.55 |
| Pentagonal + Al₂O₃ shell (10 nm) | 1.95 | 1.57 | 0.38 | 1.65 |
| Cylindrical | 1.90 | 1.55 | 0.35 | 1.81 |
| Experiment (Fig. S4) | — | — | — | 1.83 |
